# Supplementary material for: SARS-CoV-2 entry and fusion are independent of ACE2 localization to lipid rafts
Source: J Virol. 2024 Nov 21;99(1):e01823-24. doi: 10.1128/jvi.01823-24 (PMC11784143; doi:10.1128/jvi.01823-24)
Supplement: Figures S1 to S3 — Western blot, flow cytometry analysis, and raw microscopy analysis. [file jvi.01823-24-s0001.pdf]

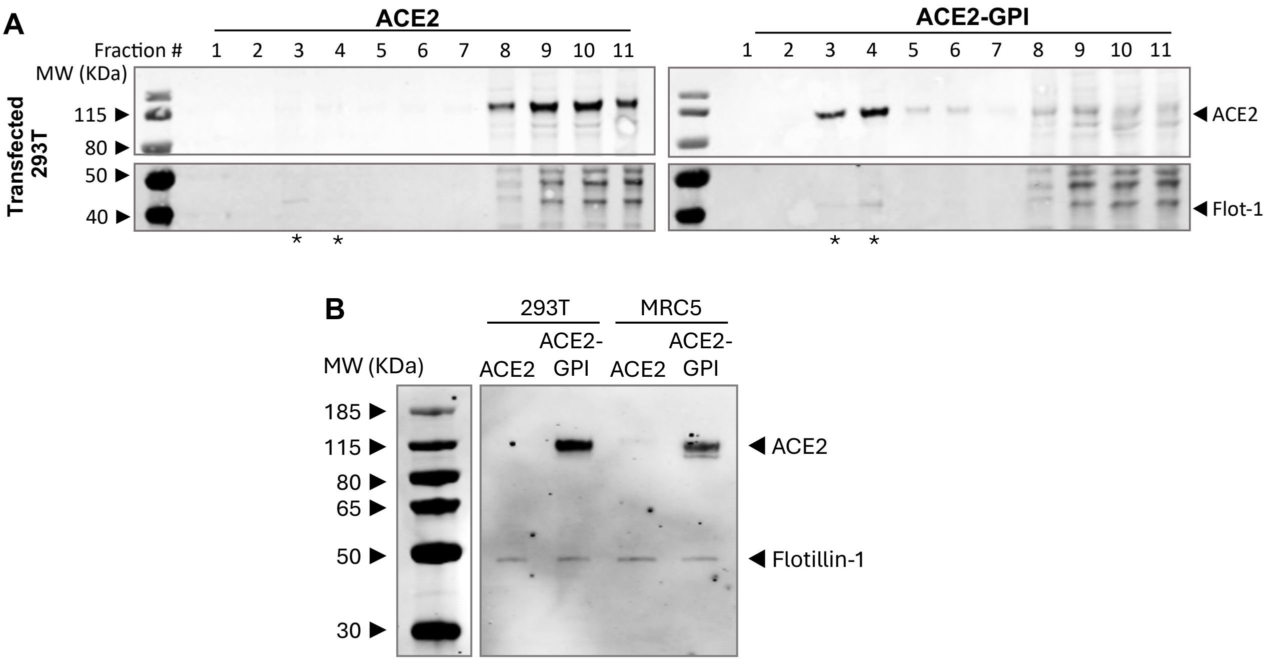

Supplementary figure 1. (A) Cell lysates of transfected 293T cells expressing ACE2 or ACE2-GPI were ultracentrifuged in an iodixanol (OptiPrep) gradient to isolate cellular membranes on their lipid content. * = Lipid raft associated protein, flotillin-1, was used as a control for lipid raft localization. **(B)** Fraction number 3 from the membrane flotation assays was taken from the 293T and MRC5 cell lines stably expressing either ACE2 or ACE2-GPI and run on a western blot. The blot was stained with anti-ACE2 and anti-flotillin-1 antibodies.

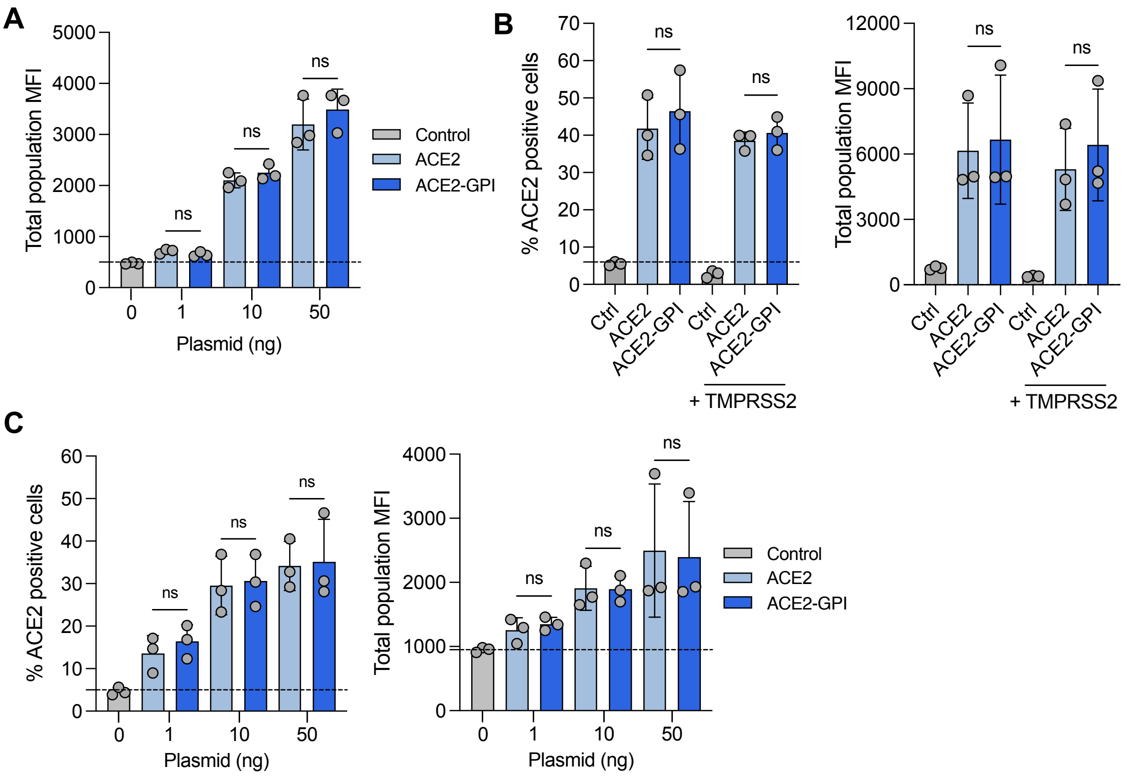

Supplementary figure 2. (A) Surface expression by MFI of ACE2 and ACE2-GPI from 293T cells in experiment from Fig. 3D. **(B)** Surface expression of ACE2 and ACE2-GPI by % ACE2 positive cells (left) and MFI (right) from 293T cells in experiments from Fig. 4F & 4G. **(C)** Surface expression of ACE2 and ACE2-GPI by % ACE2 positive cells (left) and MFI (right) from 293T cells in experiments from Fig. 5F. For A, B, C, Mann-Whitney tests were performed to compare ACE2 and ACE2-GPI conditions, ns = not significant.

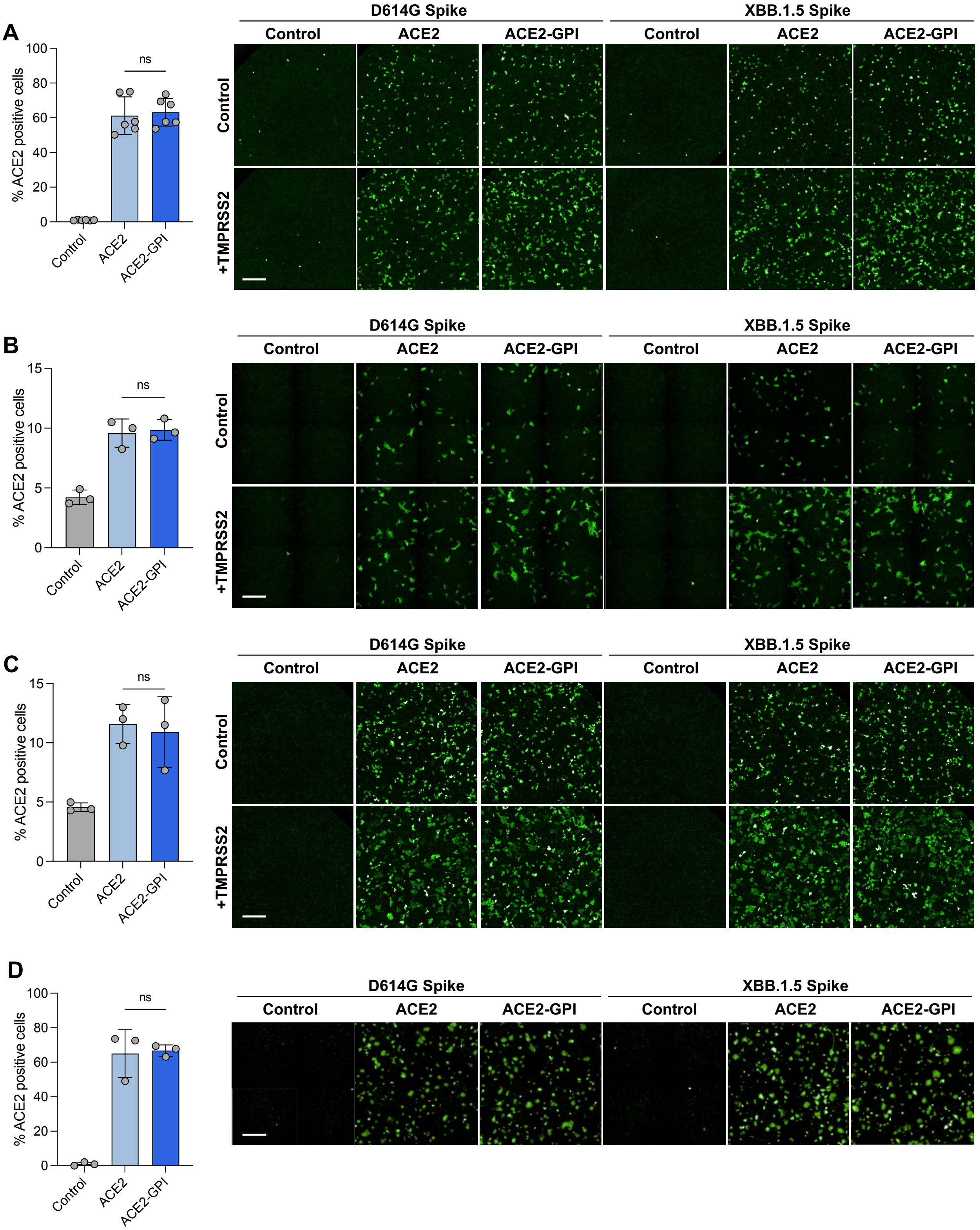

Supplementary figure 3. Cell surface expression controls measured by flow cytometry for ACE2, Spike and TMPRSS2 together with raw image data showing GFP-positive syncytia in 293T cells **(A)**, U2OS cells **(B)**, HeLa cells **(C)**, and MRC5 cells **(D)** taken from one independent replicate of each. Scale bars for A and C = 400 μm. Scale bars for B and D = 200 μm. For all panels Mann-Whitney tests were performed to compare ACE2 and ACE2-GPI conditions, ns = not significant.
